# Loss of LZAP inactivates p53 and regulates sensitivity of cells to DNA damage in a p53-dependent manner

**DOI:** 10.1038/oncsis.2017.12

**Published:** 2017-04-10

**Authors:** J J Wamsley, C Gary, A Biktasova, M Hajek, G Bellinger, R Virk, N Issaeva, W G Yarbrough

**Affiliations:** 1 Division of Otolaryngology, Department of Surgery, Yale University School of Medicine, New Haven, CT, USA; 2 Yale Cancer Center, Yale University School of Medicine, New Haven, CT, USA; 3 Department of Pathology, Yale University School of Medicine, New Haven, CT, USA

## Abstract

Chemotherapy and radiation, the two most common cancer therapies, exert their anticancer effects by causing damage to cellular DNA. However, systemic treatment damages DNA not only in cancer, but also in healthy cells, resulting in the progression of serious side effects and limiting efficacy of the treatment. Interestingly, in response to DNA damage, p53 seems to play an opposite role in normal and in the majority of cancer cells—wild-type p53 mediates apoptosis in healthy tissues, attributing to the side effects, whereas mutant p53 often is responsible for acquired cancer resistance to the treatment. Here, we show that leucine zipper-containing ARF-binding protein (LZAP) binds and stabilizes p53. LZAP depletion eliminates p53 protein independently of its mutation status, subsequently protecting wild-type p53 cells from DNA damage-induced cell death, while rendering cells expressing mutant p53 more sensitive to the treatment. In human non-small-cell lung cancer, LZAP levels correlated with p53 levels, suggesting that loss of LZAP may represent a novel mechanism of p53 inactivation in human cancer. Our studies establish LZAP as a p53 regulator and p53-dependent determinative of cell fate in response to DNA damaging treatment.

## Introduction

Despite a huge effort made in the field of targeted anticancer therapy, radiation alone, or in combination with chemotherapeutic drugs, represents one of the most powerful anticancer treatment strategies. However, acute side effects targeting the hematopoietic system often dramatically limit its application, attracting researchers to the development of (1) specific radioprotectors of normal tissue; and/or (2) drugs that radiosensitize tumors without affecting normal tissue.^1, 2^ Potential combination of both strategies in one drug must generate the optimal therapeutic window of radio- and/or chemotherapy leading to a successful treatment of cancer without causing harmful side effects. As radiation results in massive normal cell death due to p53-dependent apoptosis, one promising radioprotective tactic relays on the temporal inhibition of wild-type p53 (wtp53) activity. Small molecule p53 inhibitors, as well as genetic mouse models, confirmed the safety and efficiency of this approach.^3, 4, 5, 6, 7, 8, 9^

On the other hand, p53 is nearly always inactivated in human cancers through varied mechanisms. Mutations in the *TP53* gene are found in ~50% of all human tumors (in some types of cancer, including head and neck malignancy, the frequency of *TP53* mutations is about 90%) and are often associated with poor prognosis.^10, 11, 12, 13^ An exclusive feature of the *TP53* gene, distinguishing it from other tumor suppressors, is the type of cancer-related genetic alterations, with the majority (>80%) of them being missense point mutations resulting in the accumulation of stable mutant protein that has lost its original wild-type activity in the nucleus of tumor cells.^14, 15, 16, 17^ Many p53 mutations convey oncogenic activity that increases resistance to radiation and DNA damaging therapy, suggesting downregulation and/or inhibition of mutant p53 (mtp53) as a therapeutic strategy to enhance response to conventional chemotherapeutic drugs or radiation.^14, 16, 18, 19, 20, 21, 22, 23^ Much effort has been applied toward restoring wild-type p53 functions in mutant p53-expressing cells;^24, 25, 26, 27^ however, temporal decrease of both mutant (present in cancer cells) and wild-type (expressed in normal surrounding cells) p53 has not been extensively addressed. The strategy of simultaneous downregulation of mutant and wild-type p53 should decrease the resistance of tumors with mutant p53 to radiation and chemotherapy, while simultaneously protecting normal tissues from severe side effects.

LZAP (LXXLL/leucine zipper-containing ARF-binding protein), also known as CDK5RAP3, C53, IC53 and HSF-27, was initially identified as a binding partner of the Cdk5 activator p35.^28^ Our laboratory furthered insight into the activity of LZAP by showing that it binds alternative reading frame (ARF) to activate p53, arrest cellular proliferation and inhibit clonogenic growth.^29, 30^ Data from our laboratory and others link LZAP to a decrease in phosphorylation of its binding partners, including p38 MAPK, Chk1/2 and RelA,^31, 32, 33, 34^ that is, at least partially, explained by the ability of LZAP to enhance WIP1 phosphatase activity.^35^ However, detailed mechanisms of LZAP functions, particularly ARF-independent LZAP effects on p53, remain unclear.

Here, we show that depletion of LZAP decreased the expression of p53, regardless of p53 mutation status. Loss of LZAP promoted p53 proteasomal degradation and decreased *TP53* messenger RNA (mRNA) levels, suggesting that LZAP regulates p53 at multiple levels. LZAP activity toward p53 was independent of ARF and Wip1, but was dependent on HDM2. Consistent with these findings, LZAP and p53 protein levels linearly correlated in human non-small-cell lung cancer (NSCLC). Depletion of LZAP in cancer cells expressing wild-type p53 protected them from DNA damage-induced cell death. Importantly, loss of even one LZAP allele in normal bone marrow cells or embryonic fibroblasts derived from an LZAP heterozygous mice increased cellular resistance to DNA damage. In contrast, cancer cells expressing mutant p53 were sensitized to DNA damage by LZAP depletion. Together, these data suggest that loss of LZAP represents a new pathway for p53 inactivation in human cancers and that temporary inhibition of LZAP may be a fruitful therapeutic strategy for tumors with mutant p53.

## Results

### LZAP loss decreases p53 expression regardless of p53 mutation status

**Figure 1:**
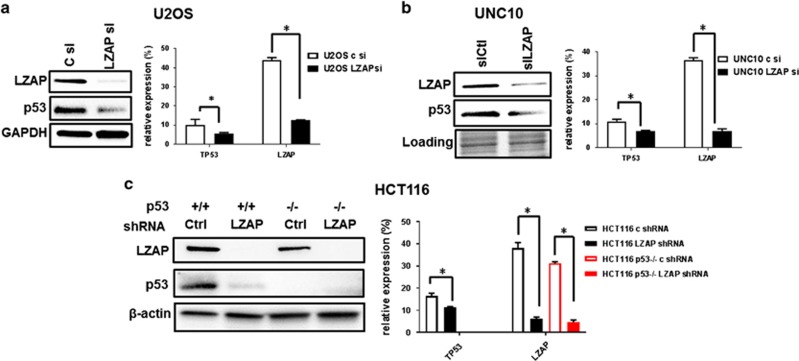
LZAP depletion results in downregulation of endogenous wild-type and mutant p53. LZAP and p53 protein (left) or relative to GPDH mRNA levels (right) in U2OS (**a**), UNC10 (**b**) or HCT116 (**c**) cells transfected with control or siRNAs specific to LZAP. mRNA levels were determined using qRT-PCR; mean from two experiments is shown, error bar represents s.d.; *P*-values were calculated using unpaired *t*-test.

We previously showed that LZAP activates p53 through both ARF-dependent and ARF-independent mechanisms. To test whether loss of LZAP could inactivate wild-type p53 in human cells, LZAP was depleted in U2OS osteosarcoma cells (Figure 1a, left) by small interfering RNA (siRNA). Downregulation of LZAP remarkably decreased p53 protein in U2OS cells. p53 is important for cellular processes such as cell cycle, differentiation, immune response, metabolism, DNA repair and senescence, and is a potent inducer of apoptosis; therefore, p53 protein levels are tightly regulated by multiple mechanisms.^36, 37^ Observed downregulation of p53 at the protein level following LZAP depletion could result from decreased *TP53* transcription, mRNA stability, translation and/or protein stability. To begin exploring these possibilities, *TP53* mRNA levels were measured by quantitative reverse transcriptase real-time PCR (qRT-PCR) with and without LZAP depletion in U2OS. LZAP downregulation decreased p53 mRNA levels in U2OS cells (Figure 1a, right).

As loss of LZAP was associated with downregulation of wtp53, we next determined if depletion of LZAP similarly affected mutant p53 protein. Endogenous mutant p53 (R248Q) was downregulated in UNC10 cells at protein (Figure 1b, left) and mRNA (Figure 1b, right) levels after transfection with LZAP, but not a control, siRNAs.

Thus, depletion of LZAP diminished p53 levels independently of p53 mutation status.

Similar to U2OS cells, depletion of LZAP decreased p53 expression in colon cancer HCT116 cells (Figure 1c).

**Figure 2:**
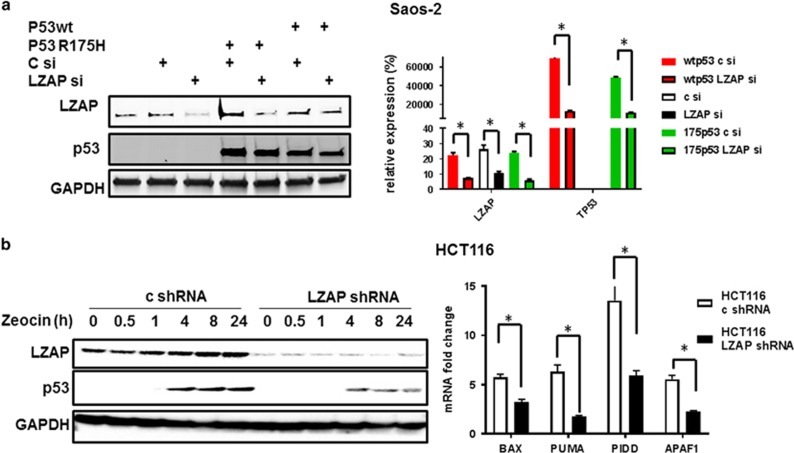
LZAP depletion downregulates exogenously expressed p53 and attenuates p53 induction and transactivation in response to DNA damage. (**a**) LZAP and ectopically expressed wild-type or mutant p53 R175H protein levels (left) or relative to GPDH mRNA levels (right) in p53 null Saos-2 cells expressing control or LZAP siRNA. mRNA levels were determined using qRT-PCR; mean from two experiments is shown, error bar represents s.d.; *P*-values were calculated using unpaired *t*-test. (**b**) Immunoblot detecting LZAP and p53 in HCT116 cells infected with retrovirus containing control or shRNA specific to LZAP prior to treatment with zeocin (200 μg/ml) for the indicated times (left); right: fold change in the expression of p53 pro-apoptotic transcriptional targets in HCT116 cells stably control or LZAP shRNA after zeocin treatment (200 μg/ml) for 24 h, as measured by qRT-PCR. Mean from three experiments is shown, error bar represents s.d.

To confirm observed association between LZAP and p53 expression levels regardless of p53 mutation status, p53 null osteosarcoma cells, Saos-2 cells were co-transfected with control or LZAP siRNAs and wild-type or mutant (R175H) p53. As found with endogenous wild-type (Figures 1a and c) and mutant (Figure 1b) p53, depletion of LZAP downregulated exogenous expression of p53 independently of mutation status (Figure 2a).

### LZAP loss inhibits p53 induction and transactivation in response to DNA damage

Following DNA damage, p53 accumulates and transactivates pro-apoptotic and growth inhibitory genes. In addition, p53 induces apoptosis through transcriptional repression of another set of genes, and through direct activation of the mitochondrial apoptotic pathway.^36, 38, 39, 40, 41^ As the transcriptional transactivation function of p53 is very important for the induction of apoptosis, we investigated whether downregulation of p53 following LZAP depletion inhibited p53 transactivation function. LZAP was depleted by short hairpin RNA (shRNA) in HCT116 cells and the cells were treated with zeocin to induce DNA damage. shRNA-induced downregulation of LZAP significantly inhibited upregulation of p53 at several time points after zeocin treatment (Figure 2b, left). Importantly, induction of p53 pro-apoptotic target genes *BAX, PUMA, PIDD and APAF1* was attenuated in cells expressing LZAP shRNA, as compared to control shRNA cells (Figure 2b, right).

Stress responsive kinases ATM and ATR are rapidly activated after DNA damage and phosphorylate p53 protein at different sites, including Ser15, leading to the disruption of the interaction between p53 and HDM2 with resultant p53 stabilization and activation.^42^ To determine whether depletion of LZAP reduced p-p53 (Ser15) level, CRISPR constructs targeting LZAP were stably transfected into U2OS cells prior to zeocin treatment. Indeed, following DNA damage, LZAP loss decreased both p-p53 (Ser15) and total p53 levels (Supplementary Figure S1).

Taken together, these data suggest that depletion of LZAP results in decreased levels of total p53 protein, as well as p53 transcriptional transactivation following DNA damage.

### Downregulation of LZAP confers resistance to DNA damage in wild-type p53-expressing cells, but renders mutant p53 cells more sensitive to a treatment

P53 is a primary regulator of cellular response to standard anticancer therapies.^36, 37, 43^ Therefore, transient suppression of wtp53 has been proposed as a protective strategy to spare normal cells consequences of treatment.^3, 4, 9, 44, 45^ On the other hand, mutations in the *p53* gene frequently have gain-of-function activity associated with increased resistance to DNA damage. Inhibition of gain-of-function p53 mutants is an attractive target for anticancer therapy, particularly in combination with radiation and chemotherapy.^19, 21, 23^ As LZAP depletion downregulated both wild-type and mutant p53 (Figures 1 and 2), we suspected that LZAP depletion may protect cells with wtp53, while sensitizing cells with mtp53 to DNA damage.

**Figure 3:**
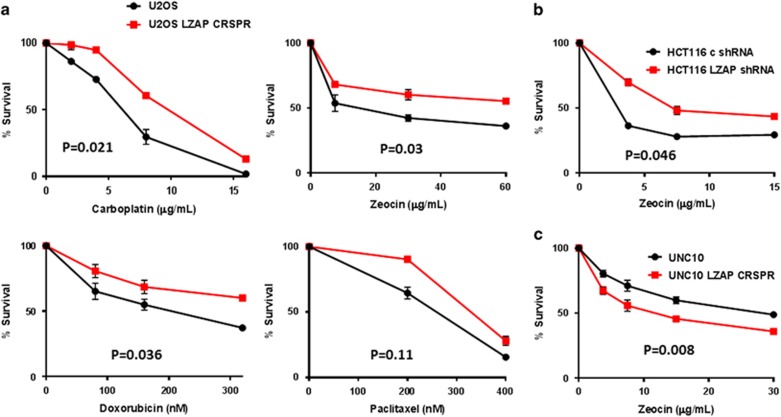
LZAP depletion protects wild-type p53-expressing cells from DNA damage, while sensitizes mutant p53 cells to the treatment. (**a**) U2OS parental and LZAP CRISPR cells were plated (1000 cells per well of 96 well plates) prior to treatment with the indicated DNA-damaging agents. Six days later, viability was measured using Cell Titer Glo (Promega). (**b**) HCT116 stable LZAP knockdown cells (or control) or UNC10 parental, or LZAP CRISPR cells (**c**) were treated with zeocin for 6 days prior to viability analysis. Mean is shown, error bars represent s.d., *N*=3; *P*-values were calculated using paired *t*-test.

To explore if the loss of wtp53 accompanying LZAP depletion protects cells from DNA damage, U2OS LZAP CRISPR and parental cells were treated with increasing doses of carboplatin (DNA/DNA and DNA/protein crosslinker), doxorubicin (DNA-intercalating agent), paclitaxel (microtubule stabilizer and anti-mitotic) and radiomimetic zeocin. Indeed, loss of LZAP protected U2OS cells from these DNA- damaging agents (Figure 3a). Similar results were observed following zeocin treatment in HCT116 LZAP shRNA cells (Figure 3b). Remarkably, in contrast to wild-type p53-harboring cells, LZAP loss in mutant p53-expressing cells UNC10 caused increased sensitivity to zeocin (Figure 3c).

Cancer cells expressing wtp53 were protected from DNA damage-induced cell death; however, potential clinical relevance relies on determining the effect of LZAP loss on normal, non-cancer cells.

**Figure 4:**
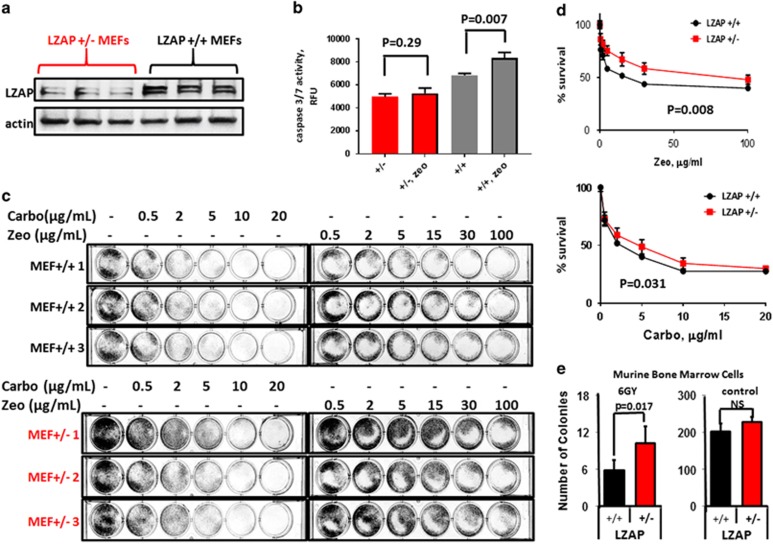
Loss of a single Cdk5rap3/Lzap allele in LZAP heterozygous mice results in increased resistance of cells to DNA damage. (**a**) LZAP protein levels in LZAP+/− and LZAP+/+ MEFs (genotyping of MEFs is shown in Supplementary Figure S2). (**b**) Caspase 3/7 activity (cleavage of the fluorescent substrate) in LZAP+/− and LZAP+/+ MEFs treated with zeocin for 6 h; experiment was performed twice in three LZAP+/− or three LZAP+/+ MEFs; *P*-values are calculated with unpaired *t*-test. (**c**) LZAP+/− and LZAP+/+ MEFs were treated with increasing concentration of carboplatin or zeocin, alive cells were visualized by methylene blue staining 7 days after the treatment. (**d**) Viability of MEFs from **c** was determined by methylene blue extraction, followed by quantification of absorbance. Percent survival is shown relative to control cells; error bars show s.e.; assays were performed in duplicate, *P*-values were calculated with paired *t*-test. (**e**) Survival of bone marrow progenitor cells derived from untreated wild-type or LZAP+/− mice or littermates treated with 6 Gy total body irradiation was determined after 7 days of *in vitro* growth. Data are presented as mean±s.d. (*n*=2 mice per group).

Because of early embryonic lethality, observed in zebrafish (before epiboly)^46^ and in mice (<3.5 days, data not shown), mice with homozygous loss of LZAP were not available for the study. However, mouse embryonic fibroblasts (MEFs) derived from LZAP+/− mice (Supplementary Figure S2) expressed lower LZAP protein levels, as compared to LZAP+/+ MEFs (Figure 4a). Zeocin treatment activated caspases in wild-type, but not in LZAP+/− MEFs (Figure 4b). Importantly, LZAP+/+ MEFs were significantly more sensitive than LZAP+/− MEFs to DNA damage induced by zeocin or carboplatin treatment (Figures 4c and d).

Bone marrow mononuclear cells are exquisitely sensitive to radiation through mechanisms largely attributed to p53-associated apoptosis. Bone marrow sensitivity is the major cause of organismal demise following whole-body irradiation, and is the major dose limiting factor for many chemotherapy regimens; however, p53 inhibition abrogates this syndrome.^7, 47^ To begin exploring the effect of LZAP loss on bone marrow cell survival after radiation, wild-type (LZAP+/+) and LZAP heterozygous (LZAP+/−) mice were irradiated with sublethal doses of total body irradiation, and clonogenic growth of isolated bone marrow mononuclear cells was determined. total body irradiation decreased colony-forming capacity in cells derived from both wild-type and LZAP+/− mice; however, bone marrow progenitor cells derived from LZAP+/− mice were significantly protected compared to cells derived from wild-type mice (Figure 4e).

These data suggest that lower LZAP expression driven by a loss of a single *Cdk5rap3/Lzap* allele in LZAP heterozygous mice is sufficient to render embryonic fibroblasts or bone marrow mononuclear cells resistant to DNA damage.

Taken together, our results demonstrate that LZAP downregulation protects cells carrying wtp53 from DNA-damaging agents, while sensitizing those with mtp53.

### Depletion of LZAP alters cellular response to DNA damage in a p53-dependent manner

**Figure 5:**
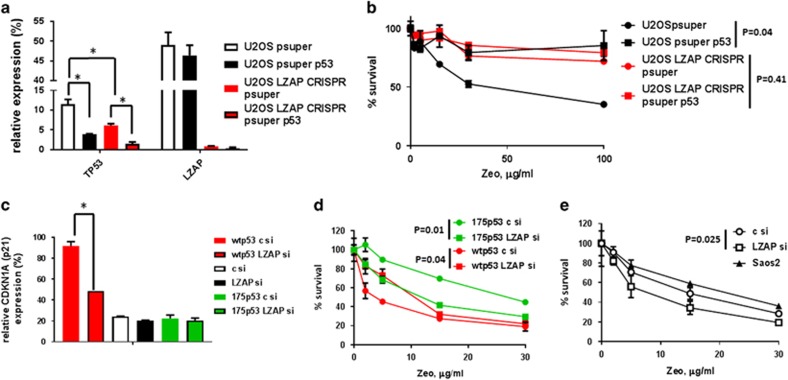
LZAP depletion affects cells survival after DNA damage in a p53-dependent manner. (**a**) Relative to GPDH mRNA levels of LZAP or TP53 in U2OS or U2OS LZAP CRISPR cells transiently transfected with psuper vectors expressing control or p53 shRNAs as determined on qRT-PCR. (**b**) Survival after increasing concentrations of zeocin of cells from **a** as determined by methylene blue staining, extraction and absorbance measurement, 7 days after the treatment. (**c**) Relative to GPDH CDKN1A expression in p53 null cells Saos-2 transfected with control or LZAP siRNAs and wild-type or mutant p53, as determined by qRT-PCR; mean from two experiments is shown, error bar represents s.d.; *P*-values were calculated using unpaired *t*-test. Survival after increasing doses of zeocin of Saos-2 cells-co-transfected control or LZAP siRNA and wild-type or mutant p53 (**d**), or Saos-2 cells transfected or not with control or LZAP siRNAs (**e**), *P*-values were calculated using paired *t*-test.

The role of LZAP in DNA damage response is well documented. It was reported that LZAP modulates the G2/M checkpoint and enhanced DNA damage-induced cell death.^32^ In addition, activation of checkpoint kinases Chk1 and Chk2 was partially inhibited by LZAP overexpression.^33^ To confirm that LZAP depletion regulated cell survival after DNA damage in a p53-dependent manner, p53 was depleted with shRNA in U2OS or U2OS LZAP CRISPR cells (Figure 5a). U2OS LZAP CRISPR cells transiently transfected with control shRNA survived significantly better than U2OS cells expressing control shRNA after zeocin treatment (Figure 5b). Depletion of p53 increased resistance of U2OS, but not U2OS LZAP CRISPR cells to zeocin (Figure 5b). In fact, survival curves after zeocin treatment of all cells with downregulated p53—U2OS p53 shRNA, U2OS LZAP CRISPR control shRNA and U2OS LZAP CRISPR p53 shRNA—were very similar (Figure 5b).

To further investigate p53-dependent effect of LZAP loss after DNA damage, p53 null Saos-2 cells were transiently transfected with control or LZAP siRNAs together with wild-type or mutant p53 R175H (Figure 2a). Elevated expression of p53 target gene *CDKN1A* (p21) in cells expressing wild-type p53 and control siRNA was diminished in cells expressing LZAP siRNA (Figure 5c). In contrast, depletion of LZAP did not change p21 expression in cells expressing mutant or no p53 (Figure 5c). Notably, Saos-2 cells expressing mutant p53 and LZAP siRNA were more sensitive to zeocin treatment than Saos-2 cells transfected with mutant p53 and control siRNA (Figure 5d). Opposite to mutant p53-expressing cells, Saos-2-wild-type p53-LZAP siRNA cells were more resistant to zeocin, as compared to Saos-2-wtp53-control siRNA cells (Figure 5d). Interestingly, we found a moderate, but significant, sensitization to zeocin in p53 null Saos-2 cells expressing LZAP siRNA compared to Saos-2 cells expressing control siRNA (Figure 5e).

These data show that LZAP depletion sensitized cells to DNA damage in the absence of p53 (Figure 5e) or in the presence of mutant p53 (Figure 5d, green), whereas LZAP depletion in cells expressing wtp53 was protective (Figure 5d, red).

Together, our experiments confirmed that the effect of LZAP depletion on cell survival after DNA damage depends on p53 status, with cells expressing wild-type p53 being protected and cells with mutant p53 or without p53 being sensitized.

### LZAP binds p53 and HDM2

**Figure 6:**
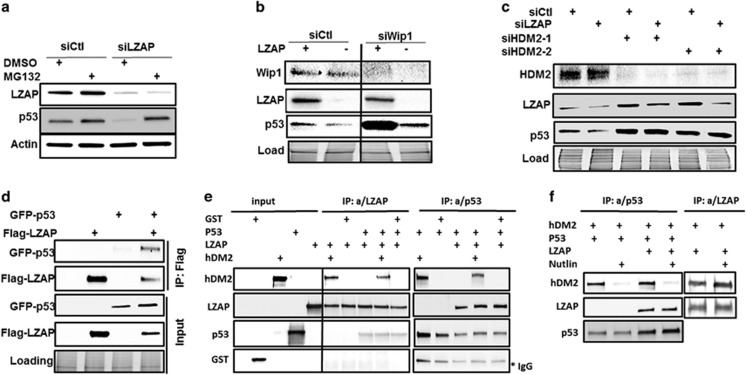
LZAP binds both p53 and HDM2. (**a**) Immunoblots of LZAP and p53 of U2OS lysates following transfection with control or LZAP siRNA, and treatment with vehicle or MG132 for 4 h. (**b**) Immunoblots of LZAP, p53 and Wip1 of U2OS (parental and LZAP CRISPR) lysates following transfection with control or Wip1 siRNA. All lanes were run on the same gel; solid line indicates where images were cropped. (**c**) Immunoblots of LZAP, p53 and MDM2 of U2OS lysates following transfection with combinations of non-targeting siRNA, LZAP siRNA or one of two siRNAs targeting MDM2. (**d**) U2OS cells were transfected with indicated plasmids encoding tagged Flag-LZAP or GFP-p53. Immunoprecipitates were prepared using Flag affinity matrix to pulldown LZAP, resolved on SDS–polyacrylamide gel electrophoresis, and immunoblotted by antibodies recognizing Flag(-LZAP) or GFP(-p53). Expression of LZAP and p53 was confirmed by immunoblotting whole-cell lysates with Flag or GFP antibodies, respectively. (**e**) Purified LZAP was incubated with p53 along or together with GST-HDM2 or GST proteins followed by pulldown with agarose beads conjugated with LZAP or p53 antibodies and detection with HDM2, LZAP, p53 or GST antibodies (see Materials and methods section). (**f**) Purified p53 was incubated with GST-HDM2 along or together with LZAP in the presence or absence of nutlin. Similarly, LZAP was incubated with GST-HDM2 in the presence or absence of nutlin, followed by pulldown with agarose beads conjugated with LZAP or p53 antibodies and detection with HDM2, LZAP or p53 antibodies.

p53 protein is thought to be turned over primarily by the 26S proteasome followed by its polyubiquitination; therefore, we examined the ability of proteasomal inhibition to reverse the decrease in p53 levels that accompany LZAP depletion. U2OS cells were transfected with either control or LZAP-specific siRNAs prior to 4-h treatment with MG132 or vehicle. Lysates harvested from cells treated with DMSO show a significant decrease in p53 protein level following LZAP depletion (Figure 6a); however, this reduction was completely reversed by inhibition of the 26S proteasome (Figure 6a). Moreover, blocking protein synthesis with cycloheximide treatment following knockdown of LZAP in U2OS cells, spotted a moderate decrease in the p53 half-life (Supplementary Figures S3A and B). These findings suggest that in addition to p53 mRNA (Figures 1 and 2), LZAP may regulate p53 at the level of protein turnover.

p53 is almost always inactivated in human cancers, either by mutation or indirectly through binding to viral proteins, or as a result of alterations in genes, whose products either activate, stabilize or carry signals from p53 including ARF, Wip1 and HDM2.^36, 37, 43, 48^ As neither U2OS nor HCT116 cells express ARF due to promoter methylation^49, 50^ (Supplementary Figure S4), LZAP regulation of p53 does not require ARF, as we previously reported. Recently, our laboratory found that LZAP binds the phosphatase Wip1,^35^ a negative regulator of p53. Wip1 dephosphorylates p53 at Ser15, resulting in its destabilization and inactivation.^51^ To determine if Wip1 was required for the regulation of p53 levels observed upon loss of LZAP, U2OS CRISPR cells were transfected with control siRNA or siRNA targeting Wip1. LZAP loss resulted in downregulation of p53 levels in the presence or absence of Wip1 (Figure 6b), suggesting that the effect of LZAP loss on p53 levels is independent of Wip1.

HDM2 is the most prominent negative regulator of p53, as indicated by its amplification and overexpression in human cancers and by p53-mediated embryonic lethality observed upon HDM2 deletion. HDM2 binds p53, inhibits its transactivation activity and directly ubiquitinates p53, ultimately leading to its proteasomal degradation.^52, 53^ Importantly, we recently reported that LZAP directly binds HDM2.^35^ To determine if HDM2 was essential for downregulation of p53 protein observed following LZAP loss, LZAP was depleted by siRNA transfection in U2OS cells in the presence or absence of two different siRNAs targeting HDM2. As expected, HDM2 depletion increased p53 levels when compared to transfection with non-targeting siRNA (Figure 6c), but it also surprisingly upregulated LZAP levels, suggesting that HDM2 may work as E3 ubiquitin ligase degrading LZAP. Expectedly, depletion of LZAP decreased p53 levels in control siRNA-expressing cells, but this effect was abrogated by HDM2 knockdown (Figure 6c).

Because LZAP regulated p53 protein stability (Figure 6a; Supplementary Figure S3), we hypothesized that LZAP may directly bind p53 to promote its stabilization. To explore possible interactions, we ectopically expressed Flag-LZAP in the presence or absence of GFP-wtp53 and immunoprecipitated LZAP with anti-Flag affinity agarose gel. Overexpression of LZAP increased GFP-p53 levels (Figure 6d, inputs), as we previously reported. GFP-p53 proteins were readily detectable in LZAP immunoprecipitates (Figure 6d). These data show that exogenously expressed LZAP and p53 interact in mammalian cells, providing a potential mechanism for LZAP’s regulation of p53 protein stability.

To confirm and further examine LZAP binding to HDM2 and p53, complex formation was investigated using recombinant proteins in cell-free system. LZAP was found to bind p53 along, GST-HDM2 along, as well both p53 and GST-HDM2, but not GST protein (Figure 6e, immunoprecipitation with LZAP antibody). Likewise, p53 was found in the complex with LZAP, GST-HDM2 or both LZAP and GST-HDM2, but not GST (Figure 6e, immunoprecipitation with p53 antibody). These results suggested that LZAP and p53 independently bind different parts of HDM2. Confirming this hypothesis, addition of specific HDM2 inhibitor nutlin^54^ disrupted the interaction between p53 and GST-HDM2, but did not influence the binding of LZAP to p53 in the same reaction (Figure 6f). Moreover, nutlin did not alter the interaction between LZAP and HDM2 (Figure 6f). These data allowed us to suggest that LZAP binds to different parts of HDM2 than p53, and that all three proteins may exist in one complex.

Taken together, our results propose that LZAP binds both HDM2 and p53, and regulates p53 levels in a HDM2-dependent manner.

### Loss of LZAP represents a new mechanism of p53 inactivation in cancer

The p53 protein does not function properly in human cancers, being inactivated directly by mutations in the *TP53* gene or indirectly by viral proteins. Alternatively, p53 function can be inhibited by alterations in genes, whose products regulate p53 itself or signaling to or from p53.^36, 37, 43^ Altered genes in human cancer that impact p53 function include, but are not limited to: amplification and overexpression of a major negative p53 regulator, HDM2;^52, 53^ loss of expression of p14ARF, a negative regulator of HDM2;^49^ overexpression of ΔNp73 (NH2-terminally truncated, transactivation-deficient, dominant-negative isoform of p53 homolog p73), which blocks p53 activities;^55, 56, 57^ mutations in tumor suppressor PTEN;^58, 59^ and disruption of Chk1/2 signaling.^60^ Our data suggest that depletion of LZAP downregulated steady-state p53 levels and inhibited radiation-induced stabilization, and activation of wild-type p53 (Figures 1 and 2). We previously reported that LZAP protein expression is decreased in ~30% of head and neck squamous cell carcinomas. These findings led us to hypothesize that loss of LZAP may represent a novel mechanism of p53 inactivation in human cancer.

**Figure 7:**
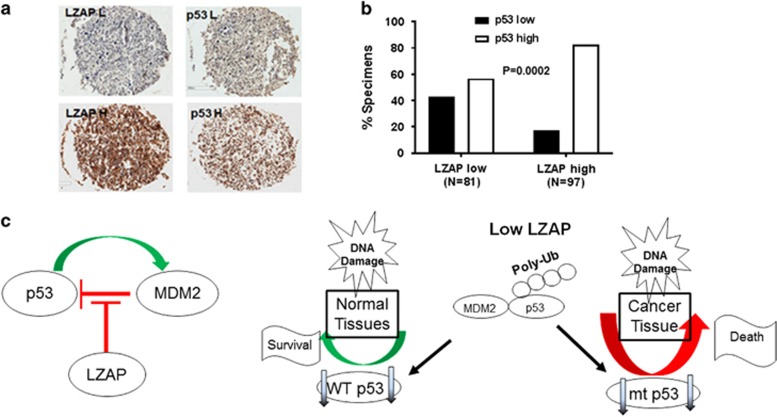
LZAP and p53 protein levels correlate in non-small-cell lung cancer. (**a**) Left: representative photomicrographs of LZAP and p53 IHC NSCLCs. (**b**) Quantification of IHC (primary NSCLC, *n*=178); high LZAP/p53 group, strong staining in >20% of tumor cells, others designated as low. The proportions of low and high p53 staining were divided based on low and high LZAP staining. *P*=0.0002 analyzed by 2 × 2 contingency table (Fisher’s two-tailed test). (**c**) Left: schematic for LZAP regulation of p53 through HDM2. When LZAP is present, it stabilizes p53. Right: LZAP loss downregulates p53 in a MDM2-dependent manner. Following DNA damage, LZAP depletion decreases wild-type p53 in normal tissues resulting in protection, while sensitizing tumor cells harboring mutant p53 to the treatment. IHC, immunohistochemistry.

To provide support for this hypothesis, human NSCLC specimens (*n*=178; Table 1) were examined to determine if decreased expression of LZAP correlated with decreased levels of p53 protein. A tissue microarray consisting of NSCLC tumors was stained with antibodies recognizing LZAP and p53. Slides were scored as ‘low’ for LZAP and p53 if fewer than 20% of tumor cells stained positively; others were designated as ‘high’ (Figure 7a). Remarkably, LZAP and p53 levels positively correlated with one another (Figure 7b). Only 18% of tumors within ‘high LZAP’ group expressed low p53 levels, whereas 44% of ‘low LZAP’ tumors had low p53 staining intensity (Figures 6b, *P*=0.0002 as analyzed by two-tailed Fisher’s test). Together, these data show that LZAP levels correlate with p53 levels in NSCLC, suggesting that LZAP may regulate p53 not only in experimental cell culture conditions, but also *in vivo* in human cancers.

## Discussion

Previously, we reported that overexpression of LZAP stabilizes p53 and increases wild-type p53 transcriptional activity.^29, 30^ In this study, we discovered that downregulation of LZAP decreased basal p53 protein levels and abrogated p53 phosphorylation, accumulation and transactivation activity, classically observed following DNA damage (Figures 1 and 2; Supplementary Figure S1). Supporting this result, p53 and LZAP protein levels correlated in primary NSCLC (Figure 7). As is typical for many new proteins that are implicated in tumorigenesis, the role of LZAP in cancer development and progression is likely to be dependent on accompanying molecular defects in the tumor, and the complicated nature of these interactions may be beginning to emerge with contradictory reports of LZAP as both an inhibitor of cancer cell growth and invasion, and a promoter of cell proliferation and metastasis. Given the importance of known LZAP-binding partners in human cancer (for example, ARF, p38, Wip1, RelA, Chk1 and Chk2) and the dearth of knowledge concerning functional regulation of LZAP through protein–protein interactions or posttranslational modifications, it is also possible that LZAP may play opposing roles in tumor promotion depending on surrounding cellular environment and/or genetic defects co-existing in the tumor. Data reported herein further support a context-dependent role for LZAP in cancer, potentially providing tumor suppressor effects by activating wild-type p53, but also oncogenic activities by stabilizing mutant p53 (Figures 1 and 2).

The most interesting finding of our studies is that LZAP depletion regulated DNA damage-induced cell death in a p53-dependent manner (Figures 3, 4, 5). Although a treatment strategy of simultaneous temporal downregulation of mutant and wild-type p53 has not been extensively explored, in theory, this approach should sensitize tumors with mutant p53 to radiation and chemotherapy and, at the same time, protect normal, wtp53 expressing, tissues. Support for this potential therapeutic strategy was provided by survival assays, revealing that depletion of LZAP in cells with wild-type p53 expression increased their resistance to DNA damage (Figures 3, 4, 5). Remarkably, loss of one *Lzap* allele in a genetically engineered mouse model increased radiation resistance of MEFs and bone marrow progenitors (Figure 4). Interestingly, control untreated LZAP+/− MEFs proliferated faster (Figure 4c, untreated wells; Supplementary Figure S5A). In contrast, LZAP CRISPR osteosarcoma U2OS cells were not as efficient in clonogenic survival as parental U2OS cells (Supplementary Figures S5B and C); similar effect was observed in other cancer cells expressing LZAP siRNA or shRNA (data not shown). The tendency of partial LZAP depletion to support proliferation of normal cells, while inhibiting survival of cancer cells, is intriguing and will warrant further investigation that will be best addressed with a conditional LZAP knockout mouse that we are creating in the laboratory.

In contrast to wild-type harboring cells, downregulation of LZAP in cells expressing mutant p53 sensitized them to radiomimetic zeocin (Figures 3c and 4d). Although we focused our study on LZAP activities toward p53, LZAP depletion increased a sensitivity of p53 null Saos-2 cells to zeocin (Figure 4e). The mechanism of how LZAP depletion potentiates p53 null cells to DNA damage-induced cell death remains to be elucidated; however, it is possible that inability of LZAP-depleted cells to arrest cell cycle progression^46^ may increase apoptosis in response to stress signals. Recently, it has been discovered that the immediate activation of p53 upon DNA damage mediates many toxic side effects, but is not required for the suppression of carcinogenesis.^61^ Therefore, efficient p53 activity is needed for tumor growth suppression during the period following recovery from DNA damage.^42^ We suggest that transient LZAP depletion or inhibition of LZAP activities toward p53 before DNA-damaging anticancer therapy could minimize p53-dependent toxicity of the treatment in normal tissues without decreasing the tumor-suppressive p53 function.

Mechanistically, we found that depletion of LZAP downregulated p53 at multiple levels. LZAP downregulation decreased wild-type or mutant TP53 mRNA by a small, but statistically significant amount (Figure 1). Moreover, exogenous expression of cytomegalovirus promoter-driven wild-type or mutant TP53 was inhibited in p53 null Saos-2 cells co-transfected with LZAP siRNA, as compared to cells co-transfected with control siRNA (Figure 2a). This results most likely indicated that LZAP regulates TP53 mRNA stability. Regulation of *TP53* mRNA expression and stability is an important step in controlling p53. *TP53* transcription is regulated by PKCσ,^62^ HOXA5,^63^ BCL6^64^ and by itself.^65^ In addition, several proteins, including RPL26,^66^ nucleolin,^66^ WRAP53,^67^ Wig-1^68^ and HuR,^69^ have been implicated in the regulation of *TP53* mRNA stability or translation. Recently, we found that LZAP binds HuR,^35^ therefore it was reasonable to hypothesize that LZAP may regulate TP53 mRNA stability and translation through HuR. However, some additional HuR targets (for example, Cyclin A) were downregulated in U2OS cells lacking LZAP expression, while others (Rb1, Myc) were not (Supplementary Figure S6). Therefore, whether LZAP regulates TP53 mRNA levels through HuR needs further experimental support.

It is believed that LZAP has no known enzymatic activity, and diminished p53 levels associated with LZAP depletion were dependent on the presence of HDM2—a major p53-negative regulator (Figures 6c and 7c, left). Given that LZAP directly binds both, p53 and HDM2 (Figures 6d–f), we propose that high levels of LZAP stabilizes p53. In this case, depletion of LZAP in the experimental conditions, or low LZAP levels found in cancers, lead to decrease of p53 protein. Observed upregulation of LZAP upon siRNA-mediated depletion of HDM2 (Figure 6c) suggests that LZAP is a target of HDM2 E3 ubiquitin ligase activity and brings another level of complexity to the LZAP-mediated regulation of p53.

In summary, our studies have identified a new mechanism of p53 inactivation in human cancer, connecting LZAP loss with downregulation of p53. LZAP depletion was found to protect normal and tumor cells expressing wild-type p53 from radiation and chemotherapeutic drugs, while sensitizing cells expressing mutant p53 to the treatment (Figure 7c, right). These findings raise important therapeutic considerations and suggest that strategies or drugs that temporarily inhibit LZAP activity toward p53 may be useful for treating p53-mutant cancers, while simultaneously protecting normal tissues from DNA-damaging therapeutic agents.

## Materials and methods

### Cell lines, transfection and retroviral infection

Human cell lines U2OS, Saos-2 and Phoenix were obtained from Yue Xiong in 1998 (University of North Carolina). The UNC10 cell line was created by David Witsell in 1997 (University of North Carolina). Cells were cultured in complete growth media recommended by the American Type Culture Collection (ATCC) at 37 °C in 5% CO_2._

Non-targeting siRNA (Origene, Rockville, MD, USA), siHDM2-1 and siHDM2-2 (Origene), siLZAP-1 and siLZAP-2 (Dharmacon, Lafayette, CO, USA), and siWip1 (Dharmacon) were transfected using Lipofectamine RNAiMAX (ThermoFisher, Waltham, MA, USA) as per manufacturer’s instructions. For shRNA-mediated knockdown of LZAP in HCT116 cells, the LZAP-1 siRNA sequence was inserted into the pRetro-Super retroviral vector. Control and shLZAP constructs were transfected into Phoenix cells, and supernatant containing viral particles was harvested. Stable cell lines were generated by infecting with retrovirus and selecting with puromycin (InvivoGen, San Diego, CA, USA) followed by clonal expansion. Stable LZAP CRISPR clonal cell lines were created by transfection with CRISPR constructs (Santa Cruz Biotechnology, Dallas, TX, USA) targeting LZAP and selection with puromycin.

Transfections of plasmid DNA were performed using Fugene 6 (U2OS) (Promega, Madison, WI, USA) or Lipofectamine 2000 (Phoenix; ThermoFisher) as per manufacturer’s protocol. The total amount of DNA (and siRNA) transfected was kept equal by adding appropriate amounts of empty vector (pcDNA3.1) (or non-targeting siRNA). pcDNA3-Flag-LZAP and pcDNA3-Myc3-LZAP were cloned as previously described. GFP-p53-expressing vectors, as well as psuper and psuper p53 plasmids, were a gift from G Selivanova (Sweden).

### Antibodies and reagents

Primary antibodies for immunoblotting include Flag (Sigma, St Louis, MO, USA; M2); phospho-p53 (Ser15) (Cell Signaling, #9284; Danvers, MA, USA); and GAPDH (FL-335), β-actin (N-21), GFP (B-2), Myc (9E10), Wip1 (F-10), MDM2 (SMP14 and 2A10) and p53 (DO1 and FL393) (Santa Cruz Biotechnology). LZAP custom antiserum was previously described. Other reagents include normal IgG (Promega), HRP-conjugated secondary antibodies (Promega), goat anti-mouse IgG (H+L) secondary antibody (Dylight 550 conjugate) and goat anti-rabbit IgG (H+L) secondary antibody (DyLight 650 conjugate) (ThermoFisher), zeocin (Invitrogen, Carlsbad, CA, USA), MG132 (Sigma), carboplatin (Sigma), doxorubicin (Selleckchem, Houston, TX, USA) and paclitaxel (Sigma), nutlin (Santa Cruz Biotechnology).

### Recombinant proteins

Recombinant human GST-HDM2 and recombinant human p53 proteins were from R&D systems (Minneapolis, MN, USA). GST was from Abcam (Cambridge, MA, USA). Recombinant LZAP was purified from *Escherichia coli* BL21(DE3) as described in Wamsley *et al.* ^35^

### Immunoprecipitation and immunoblotting

For immunoprecipitation, cells were lysed in RIPA buffer (Sigma) supplemented with Complete Mini EDTA-free Protease Inhibitor cocktail (Roche, Basel, Switzerland) and PhosStop (Roche). About 200 μg of lysates were pre-cleared for 30 min using normal mouse or rabbit IgG (Promega) and 20 μl protein A/G beads (Santa Cruz Biotechnology) prior to incubation with agarose beads conjugated to antibodies recognizing Flag- or Myc-conjugated beads (Sigma, 15 μl). Immunoblotting was performed as described previously .

About 100 ng of indicated recombinant proteins (Figures 6e and f) were incubated in phosphate-buffered saline supplemented with Complete Mini EDTA-free Protease Inhibitor cocktail (Roche), 0.05% Triton X100, 100 mm of NaCl and 0.05% bovine serum albumin for 1 h at 4°C. About 20 μl of DO1 (p53 antibody) conjugated to agarose (Santa Cruz Biotechnology, sc-126 AC) or 20 μl of LZAP custom antiserum conjugated to Protein A/G agarose (Santa Cruz Biotechnology) were added and incubated overnight at 40°C. The beads were spun down at 3000 r.p.m. for 1 min, washed four times with phosphate-buffered saline supplemented with 0.05% Triton X100 and 100 mm of NaCl, resuspended in 2 × Laemmli sample buffer (Bio-Rad, Hercules, CA, USA) and boiled for 3 min. Immunoblotting was performed as described.^70^

### Caspase activity

This assay was performed using Caspase 3 Activity Assay Kit (Cell Signaling, #5723) according to manufacture instructions.

### Cell viability assays

Cell viability assays were performed using Cell Titer Glo (Promega) as previously described.^46^

Alternatively, 10 000 cells per well were plated in 24 well plates, treated with indicated drugs the next day and stained with 0.5% methylene blue in methanol after 6–8 days. Pictures were taken or/and the dye was extracted from stained cells with 3% HCl solution for absorbance quantitation.

### Creation of LZAP heterozygous mice

LZAP was targeted in murine embryonic stem cells by homologous recombination using a LZAP floxed construct targeting the first two exons of murine LZAP. After selection, clones were screened by PCR and Southern blotting with two independent recombinant clones (2A2 and 2G5) identified. Mice were crossed with B6.FVB-Tg (EIIa-cre) C5379Lmgd/J mice (Jackson Laboratories, Bar Harbor, ME, USA) and then crossed for six generations with C57Bl/6 mice (Jackson Laboratories). Genotype of mice was confirmed by PCR; see Table 2 for oligonucleotide sequences. Studies have been approved by the Yale Institutional Animal Care and Use Committee.

### Primary cultures of mouse embryonic fibroblasts

MEFs were isolated from 12 days postcoitus embryos by breeding LZAP+/− females and males. The embryos were individually trypsinated in 0.05% trypsin (Invitrogen), plated and cultured in DMEM supplemented with 10% fetal bovine serum penicillin/streptomycin. Passage 2 or 3 MEFs were used in the assays.

DNA was isolated from each culture using the Qiagen DNA purification kit. The genotype of the embryos was determined by PCR using primers from Table 2.

### Bone marrow mononuclear cell colony-forming assay

C57Bl/6 wild-type and LZAP heterozygous mice were treated with total body irradiation (6 Gy) or left untreated (four mice in each group). Four hours after total body irradiation, bone marrow mononuclear cells were isolated from femurs and tibias of each mouse and plated (4 × 10^4^ cells/ml in 35 mm diameter plates) in MethoCult M3231 medium (StemCell Technologies, Vancouver, BC, Canada) supplemented with 10 ng/ml recombinant mouse GM-CSF (StemCell Technologies) and IMDM growth medium (Invitrogen). Colony formation (megakaryocyte erythrocyte progenitor (MEP) and granulocyte-monocyte progenitor (GMP)) was scored after 7 days of culture at 37 °C in the presence of 5% CO_2_. Data are analyzed using a one-tailed Student’s *t*-test.

### Quantitative reverse transcriptase real-time PCR

Total RNA was extracted from the cells using RNeasy Mini Kit (Qiagen, Hilden, Germany) and reverse transcribed into cDNA using the iScript cDNA Synthesis kit (Bio-Rad). qRT-PCR was performed using iQ SYBR Green Supermix (Bio-Rad) and CFX96 Real-Time System (Bio-Rad); see Table 2 for oligonucleotide sequences used. The expression of mRNA of interest was normalized to the expression of GPDH.

### Immunohistochemistry

Specimens were fixed with 10% formalin and embedded in paraffin per routine of the surgical pathology division. Sectioning and immunostaining were performed by the Yale Tissue Microarray Core using antibodies recognizing LZAP (HPA022141, Sigma) and p53 (DO7, Santa Cruz Biotechnology). Informed consent was obtained from each subject, and human investigations were performed after approval by an institutional review board.

## Figures and Tables

**Table 1:** Patient characteristics

*Characteristics*	*Total*	*LZAP low*	*LZAP high*	P-value
Number of cases	178	81 (45.5%)	97(55.5%)	
				
*Gender*				0.88
Male	86 (48.3%)	38 (46.9%)	48 (49.5%)	
Female	91 (51.1%)	42 (51.9%)	49 (50.5%)	
NA	1 (0.56%)	0	1 (1.2%)	
				
*Age at Dx*				0.34
Median	63	66	64	
Range	35–89	42–89	35–83	
Mean	62.4	65.8	64.3	
				
*Staging*				
*T*				
T1–T2	53 (29.8%)	26 (32.1%)	27 (27.8%)	1.00
T3–T4	19 (10.7%)	10 (12.3%)	9 (9.3%)	
NA	106 (59.6%)	45 (55.6%)	61 (62.9%)	
*N*				
N0	34 (19.1%)	18 (22.2%)	16 (16.5%)	0.25
N1–3	42 (23.6%)	16 (19.8%)	26 (26.8%)	
NA	102 (57.3%)	47 (58.0%)	55 (56.7%)	
*Stage*				
I–II	124 (69.7%)	62 (76.5%)	62 (63.9%)	0.10
III–IV	53 (29.8%)	19 (23.5%)	34 (35.1%)	
NA	1 (0.56%)	0	1 (1.2%)	

Abbreviations: LZAP, leucine zipper-containing ARF-binding protein; NA, not available.

Characteristics of patients whose tumors were included in the tissue microarray. Statistical analyses of patient groups were conducted by two-tailed Student’s *t*-test or Fisher’s exact test.

**Table 2:** Oligonucleotide sequences: oligonucleotides for confirming LZAP mice genotype (5′-TGTGCCACCACGCAACTTTT-3′ 5′-CATGAAGACAGAACCAAAC-3′)

*qRT-PCR oligonucleotides*	
*Gene*	*Sequence (5*′*-3*′*)*
*H. sapiens CDK5RAP3* F	CAATGCTGCCATCCAGGACATG
*H. sapiens CDK5RAP3* R	ATCCGCTGTGAAGAGTATCGGC
*H. sapiens TP53* F	CCTCAGCATCTTATCCGAGTGG
*H. sapiens TP53* R	TGGATGGTGGTACAGTCAGAGC
*H. sapiens CDKN1A* F	AAGACCATGTGGACCTGT
*H. sapiens CDKN1A* R	GGTAGAAATCTGTCATGCTG
*H. sapiens PPM1D* F	GAAGAAACTGGCGGAATGG
*H. sapiens PPM1D* R	TTGTGAGTGAGTCGAGGTCGT
*H. sapiens BAX* F	TGGAGCTGCAGAGGATGATTG
*H. sapiens BAX* R	GAAGTTGCCGTCAGAAAACATG
*H. sapiens APAF1* F	GCTCTCCAAATTGAAAGGTGAAC
*H. sapiens APAF1* R	ACTGAAACCCAATGCACTCC
*H. sapiens BBC3* F	GCAGGCACCTAATTGGGCT
*H. sapiens BBC3* R	ATCATGGGACTCCTGCCCTTA
*H. sapiens PIDD* F	TCTGACACGGTGGAGATGTTCG
*H. sapiens PIDD* R	AGGTGCGAGTAGAAGACAAAGCAG

Abbreviations: *H. sapiens*, *homo sapiens*; LZAP, leucine zipper-containing ARF-binding protein; qRT-PCR, quantitative reverse transcriptase real-time PCR.

Oligonucleotide sequences used for confirming mouse genotype and for qRT-PCR.
