# Supplementary material for: Loss of LZAP inactivates p53 and regulates sensitivity of cells to DNA damage in a p53-dependent manner
Source: Oncogenesis. 2017 Apr 10;6(4):e314–. doi: 10.1038/oncsis.2017.12 (PMC5520489; doi:10.1038/oncsis.2017.12)
Supplement: Supplementary Information [file oncsis201712x1.pdf]

Supplementary Figure Legends.

Figure S1. Immunoblot detecting LZAP, p53 (pSer15), and total p53 in U2OS cells stably transfected with CRISPR/Cas9 constructs targeting LZAP, before and after zeocin stimulation (200 ug/mL) for eight hours.

Figure S2. Genotyping of MEFs. MEFs used in the experiments are labelled.

Figure S3. (A) Immunoblot detecting LZAP and p53 in U2OS cells transfected with control or LZAP siRNAs and treated with cycloheximide (CHX) for indicated time points (minutes). (B) Quantification of p53 protein levels (% from 0 min) from immunoblot in (A).

Figure S4. Arf protein levels in U2OS or Saos-2 cells.

Figure S5. (A) Quantification of survival of LZAP +/- or LZAP +/+ MEFs that was determined by methylene blue extraction, followed by quantification of absorbance. (B) Clonogenic survival of U2OS or U2OS LZAP CRISPR cells. (C) Colonies from (B) were counted, and the number of colonies from U2OS LZAP CRISP cells was normalized to U2OS cells.

Figure S6. Relative to GPDH expression of several HuR targets in U2OS or U2OS LZAP CRISPR cells as determined by qRT-PCR.

S1

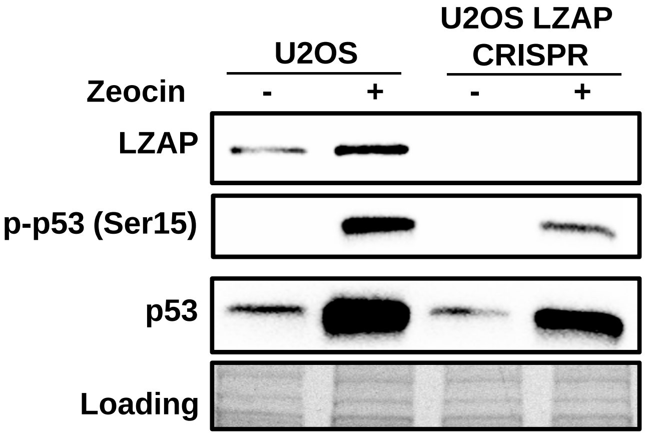

S2

A

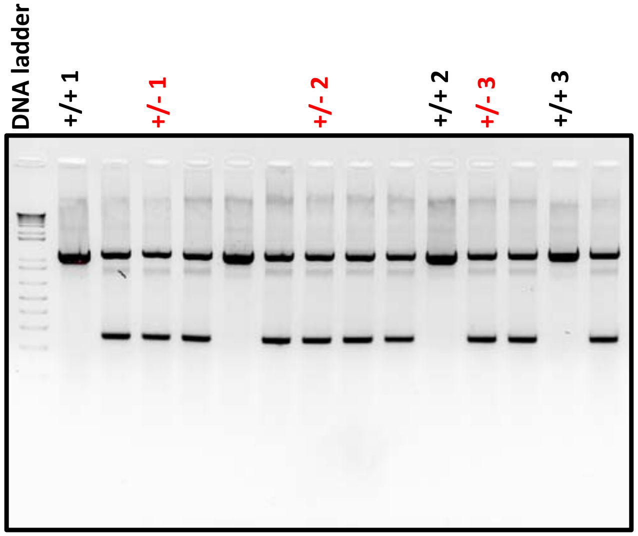

S3

A

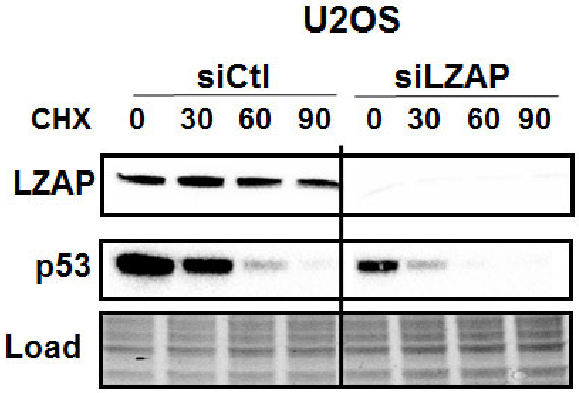
U2OS
siCtl
siLZAP
CHX 0 30 60 90 0 30 60 90
LZAP
p53
Load

B

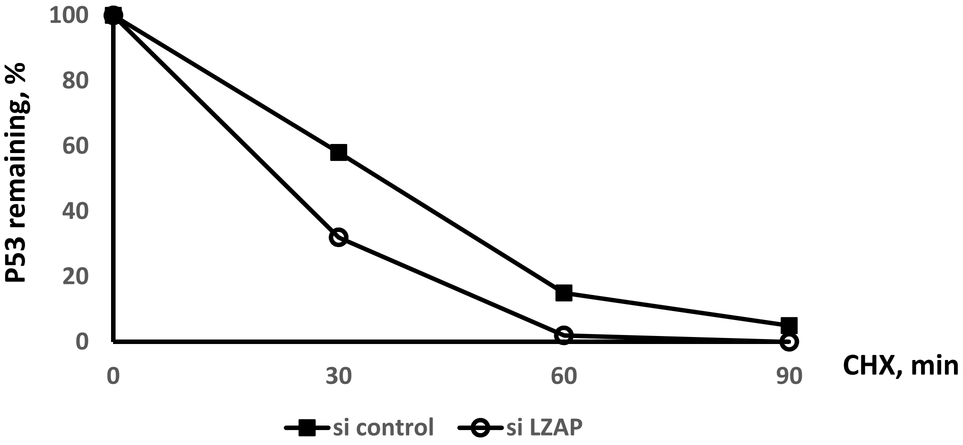
100
80
60
40
20
0
P53 remaining, %
0
30
60
90
CHX, min
si control
si LZAP

S4

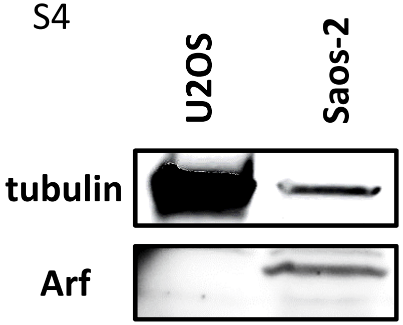

S5

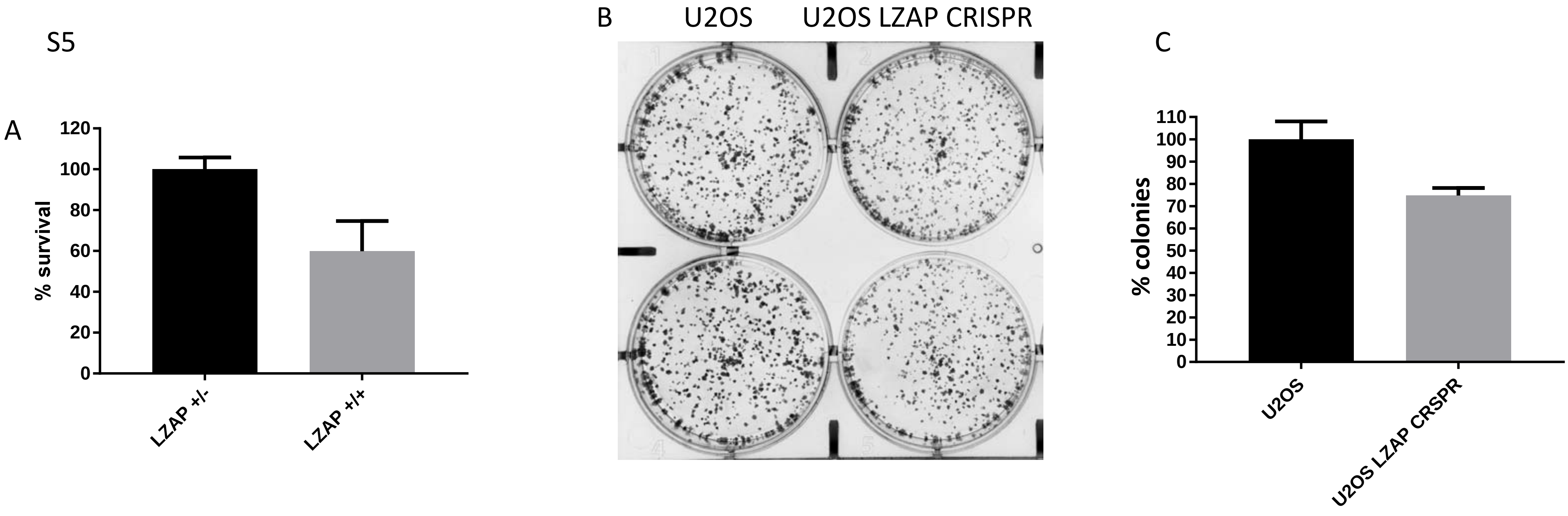

A
120
100
80
60
40
20
0
% survival
LZAP +/-
LZAP +/+
B
U2OS
U2OS LZAP CRISPR
C
110
100
90
80
70
60
50
40
30
20
10
0
% colonies
U2OS
U2OS LZAP CRSPR

S6

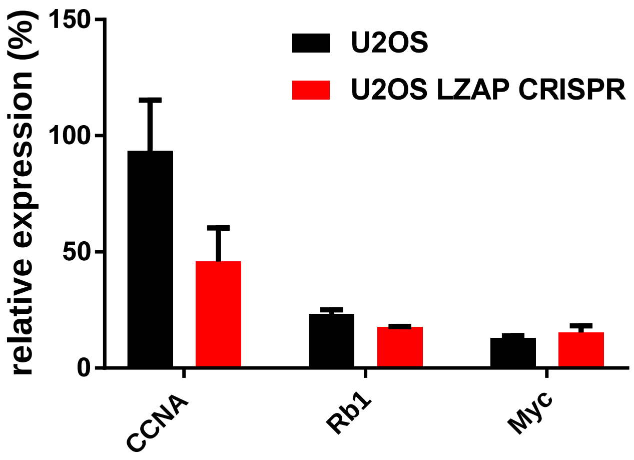
relative expression (%)
150
100
50
0
U2OS
U2OS LZAP CRISPR
CCNA
Rb1
Myc
